# miRNA as a Modulator of Immunotherapy and Immune Response in Melanoma

**DOI:** 10.3390/biom11111648

**Published:** 2021-11-08

**Authors:** Mai-Huong Thi Nguyen, Yueh-Hsia Luo, An-Lun Li, Jen-Chieh Tsai, Kun-Lin Wu, Pei-Jung Chung, Nianhan Ma

**Affiliations:** 1 Department of Biomedical Sciences and Engineering, National Central University, Taoyuan 320317, Taiwan; nguyenthimaihuong1989@gmail.com (M.-H.T.N.); t982020@gmail.com (A.-L.L.); ndmc6217316@gmail.com (K.-L.W.); charlie850729@gmail.com (P.-J.C.); 2 Department of Life Sciences, National Central University, Taoyuan 320317, Taiwan; yhLuo@g.ncu.edu.tw; 3 Institute of Biotechnology, National Tsing Hua University, Hsinchu 300044, Taiwan; b993090010@gmail.com; 4 Institute of Biotechnology and Pharmaceutical Research, National Health Research Institutes, Miaoli 350401, Taiwan; 5 Division of Nephrology, Department of Internal Medicine, Taoyuan Armed Forces General Hospital, Taoyuan 325208, Taiwan

**Keywords:** immunotherapy, resistance, miRNA, biomarker

## Abstract

Immune checkpoint inhibitors are a promising therapy for the treatment of cancers, including melanoma, that improved benefit clinical outcomes. However, a subset of melanoma patients do not respond or acquire resistance to immunotherapy, which limits their clinical applicability. Recent studies have explored the reasons related to the resistance of melanoma to immune checkpoint inhibitors. Of note, miRNAs are the regulators of not only cancer progression but also of the response between cancer cells and immune cells. Investigation of miRNA functions within the tumor microenvironment have suggested that miRNAs could be considered as key partners in immunotherapy. Here, we reviewed the known mechanism by which melanoma induces resistance to immunotherapy and the role of miRNAs in immune responses and the microenvironment.

## 1. Introduction

Melanoma is the major skin cancer-related cause of death. The survival rate of meta-static melanoma is approximately 10–15%, even though many effective approaches, such as targeted therapy and immunotherapy, have gained the approval by the Food and Drug Administration (FDA) for the treatment of melanoma. Immunotherapy leads to praiseworthy benefits and improves overall survival by approximately 35–50% for the treatment of melanoma [1]. In the past decade, different kinds of immunotherapies have been developed. Interferon (IFN)-alpha and interleukin-2 are the early immunotherapies for advanced-stage melanoma patients [2,3,4]. However, the severe toxicity and a low percentage of long-term complete response have been observed [4]. Subsequent immunotherapy approaches have focused on monoclonal antibodies targeting immune checkpoint proteins. The first immune checkpoint inhibitors were anti-cytotoxic T-lymphocyte-associated antigen 4 (CTLA-4) agents, ipilimumab and tremelimumab. However, only ipilimumab was approved by the FDA in 2011 for melanoma treatment [5,6]. The anti-programmed death 1 (PD-1) agents, nivolumab (monotherapy or in combination with ipilimumab) and pembrolizumab (monotherapy), and the anti-programmed death ligand 1 (PD-L1) agent, atezolizumab, were rapidly developed and approved by the FDA [7,8,9,10]. The combination of ipilimumab and nivolumab has been shown to be more effective than single agents [11]. CTLA-4 and PD-1 are inhibitory receptors on activated T cells, and PD-L1 is a PD-1 ligand on tumor cells. Although immunotherapy has improved the clinical outcomes, approximately 50% of melanoma patients do not respond or acquire resistance to immune checkpoint inhibitors [8,12].

miRNAs are noncoding RNAs that regulate both transcription and translation. miRNAs modulate mRNAs by targeting 3′UTR of genes. Abnormal expression of miRNAs is commonly found in cancers. It has been shown that miRNAs mediate not only the biological functions of tumor cells but also of immune cells. Recent studies have reported the modulation of miRNAs in innate and adaptive immunity by regulating the differentiation and activation of immune cells [13]. Moreover, immunosuppressive tumor microenvironment-regulated miRNAs are related to overall survival in melanoma patients [14]. It has been shown that miR-155 is involved in the activation or differentiation of immune cells including B cells, T cells, dendritic cells (DCs), natural killer (NK) cells, myeloid cells, and macrophages [15,16,17]. Some clinical trials involving certain miRNAs, such as miR-16 (NCT02369198), miR-29 (NCT03601052), and miR-34 (NCT01829971), or miRNA inhibitors, such as anti-miR-21 (NCT03373786), anti-miR-92a (NCT03603431), and anti-miR-122 (NCT01200420), have been conducted. Notably, a clinical trial of cobomarsen, an inhibitor of miR-155 (NCT02580552), has been designed to treat patients diagnosed with cutaneous T-cell lymphoma, chronic lymphocytic leukemia, diffuse large B-cell lymphoma, or adult T-cell leukemia/lymphoma. These findings indicated that miRNAs play a central role in tumor progression and the tumor microenvironment.

Emerging evidence has elucidated the contribution of miRNAs to the immune response and therapeutic options in cancers. Therefore, it is necessary to understand the biological roles of miRNAs in tumor immunity. In this review, we discuss the resistance mechanism of melanoma to immunotherapy and the involvement of miRNAs in the response to immune checkpoint inhibitors and the tumor microenvironment.

## 2. Mechanism of Immunotherapy Resistance in Melanoma

Despite an initial response to immunotherapy at the beginning, a subset of melanoma patients develop resistance. Several studies have been focused on the resistance mechanism of cancers to immune checkpoint inhibitors. In general, immunotherapy resistance was categorized as primary resistance or intrinsic resistance and acquired resistance. A nonresponsive tumor to immunotherapy was defined as primary resistance, while acquired resistance represented a tumor that initially responded to immunotherapy, but then developed resistance after a period of treatment [18]. Gene mutations and gut microbiota play an important role in the response of melanoma to immune checkpoint inhibitors.

**Figure 1:**
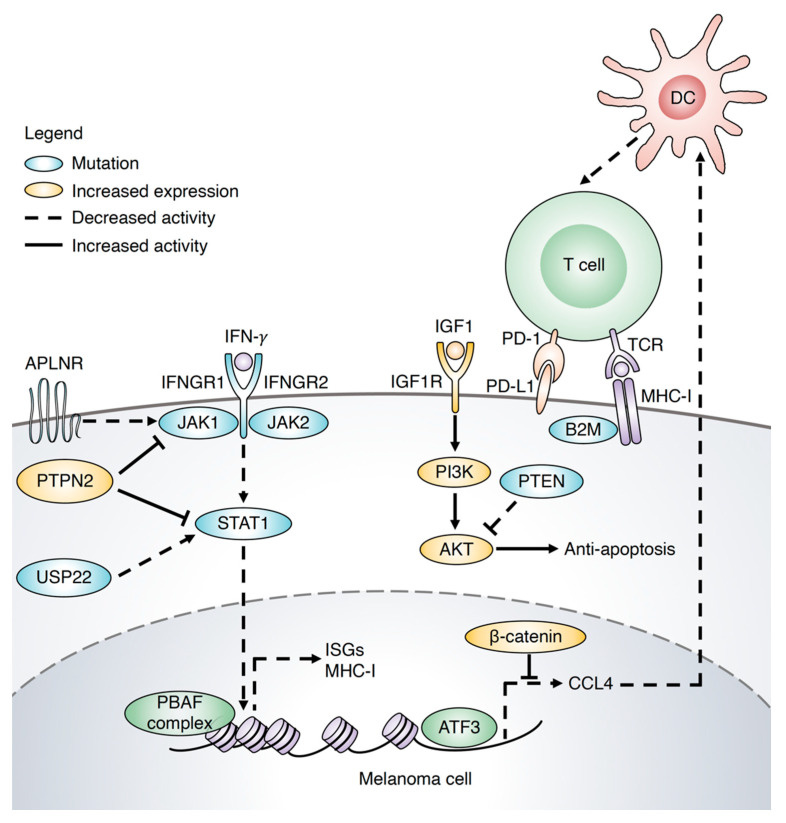
Diverse mechanism supporting resistance to immunotherapy in melanoma. JAK-STAT is a major pathway involved in resistance to immune checkpoint inhibitors in melanoma. In the normal response, T cell-derived IFN-γ binds to its receptor on the tumor cells, IFNGR1/2, and then activates the JAK-STAT pathway to induce the transcription of downstream genes including ISGs and MHC-I. CCL4 is induced by the transcription factor ATF3 to activate DC, and PTEN regulates the activity of the PI3K/AKT pathway to induce apoptosis. Mutations in JAK1/2, IFNGR1 or regulators of the JAK-STAT pathway, including APLNR and USP22, or increases in the negative modulator of JAK-STAT pathway, PTPN2, cause the inactivation of the JAK-STAT pathway and resistance to immunotherapy [19,20,21,22,23]. The PBAF complex reduces chromatin accessibility, which decreases the transcription of ISGs [24]. Active β-catenin by the WNT/β-catenin signaling suppresses CCL4 transcription from ATF3 leads to inactivation of DCs thereby reducing cytotoxic T cells [25]. Anti-apoptosis is caused by activation of PI3K/AKT due to PTEN mutation [26,27]. B2M mutation leads to unstabilization of MHC-I on the surface of tumor cells, which reduces the activation of T cells [19].

In the normal response, T cell-derived Interferon gamma (IFN-γ) binds to its receptor on the tumor cell, IFNGR1/2, then activates Janus kinase–signal transducer and activator of transcription (JAK-STAT) to induce the transcription of downstream genes including interferon-stimulated genes (ISGs) and major histocompatibility complex class I (MHC-I). In addition, CCL4 is induced by transcription factor, activating transcription factor 3 (ATF3), to activate DCs and phosphatase and tensin homolog (PTEN) regulates the activity of the PI3K/AKT pathway to induce apoptosis. However, these signaling pathways are altered by gene mutations that occur in immunotherapy resistance in melanoma (Figure 1).

Gene mutations are the key mediators of resistance of melanoma to immune checkpoint inhibitors. Mutations in Janus kinase 1/2 (JAK1/2) and beta-2-microglobulin (B2M) were found in both primary and acquired resistance to anti-PD-1 antibody (pembrolizumab) or anti-CTLA-4 (ipilimumab) in metastatic melanoma patients [19,20,28]. JAK1/2 mutations caused a defective response to IFN-γ, while B2M mutation led to a reduction in the surface expression of MHC-I. IFN-γ signaling is a key regulator of T cell activation for the antitumor response. A lack of IFN-γ pathway genes was found in the nonresponders to anti-CTLA-4 therapy (ipilimumab), and inactivation of the IFN-γ receptor, IFNGR1, caused the resistance to anti-CTLA-4 treatment in mice with melanoma [29].

Recently, several studies have undertaken a genetic screening approach by using T cell-based CRISPR-Cas9 to analyze the mechanism of immunotherapy resistance. Lack of signal transducer and activator of transcription 1 (STAT1), JAK1, or IFNGR1 in BRAFV600E/PTEN-/- mice increased tumor growth and decreased survival after anti-PD-1 treatment [21]. Notably, deletion of protein tyrosine phosphatase nonreceptor type 2 (PTPN2), a negative modulator of STAT1/JAK1 signaling, enhanced the efficiency of immunotherapy in melanoma tumors by increasing the activation of CD8+ T cells and the IFN-γ signaling pathway. Another study reported that mutation in apelin receptor (APLNR) caused resistance [22]. The IFN-γ response was positively regulated by the interaction between APLNR and JAK1. Of note, loss of APLNR decreased the benefit of anti-CTLA-4 in vivo. Inactivation of three genes, including polybromo 1 (PBRM1), AT-rich interaction domain 2 (ARID2), and bromodomain containing 7 (BRD7), that code for PBAF, one of the chromatin-remodeling complexes of the SWI/SNF, sensitized melanoma cells for destruction by T cells [24]. PBAF-deficient tumor cells efficiently responded to IFN-γ by enhancing chemokines, including CXCL9 and CXCL10, which led to the accumulation of effector T cells. Importantly, resistant tumors were sensitive to immunotherapy after inactivation of PBRM1 by increasing DCs, M1 macrophages, T cells, and NK cells. In addition, low expression of ARID2 prolonged the survival of melanoma patients with high T cell infiltration. Inactivation of ubiquitin-specific peptidase 22 (USP22), a deubiquitinating protease, reduced the sensitivity of melanoma cells to T cell-mediated killing by regulating the IFN-JAK1-STAT1 signaling pathway [23]. USP22 maintained the stability of STAT1 through regulation of deubiquitination of STAT1.

It was reported that loss of phosphatase and tensin homolog (PTEN) was associated with resistance to immunotherapy [26,27]. An analysis of metastatic melanoma patients treated with anti-PD-1 agents (pembrolizumab and nivolumab) showed that a lack of PTEN in melanoma patients increased tumor growth as compared to the PTEN-expressing patients by decreasing T cell functions [27]. Additionally, a PI3Kβ inhibitor enhanced the inhibitory effects of anti-PD-1 treatment in vivo. Of note, a recent study involved the collection of tumor samples from a metastatic melanoma patient over nine years. The results demonstrated that deletion of chromosome 15q, including B2M, caused loss of PTEN and cyclin-dependent kinase inhibitor 2A (CDKN2A) homozygous deletion was observed in the resistance to immunotherapy in melanoma patients [30].

The lack of T cell infiltration and activation of the WNT/β-catenin pathway was involved in the resistance to anti-PD-L1/anti-CTLA-4 antibody therapy in a melanoma mouse model [25,26]. The activation of β-catenin reduced the accumulation of DCs because of defective production of CCL4, which was regulated by ATF3 [25].

It was reported that high expression of sphingosine kinase-1 (SK1), an important modulator of antitumor immunity, reduced the survival of melanoma patients (1 of mucosal, 30 of cutaneous, and 1 of other subtype) after anti-PD-1 treatment [31]. SK1-downregulated mice were more sensitive to anti-CTLA-4 or anti-PD-1 treatment than were control mice as a result of decreased infiltration of regulatory T (Treg) cells and rising CD8/Treg ratio.

The interaction between melanoma cells and the immune system is influenced by the genetic alteration of human leukocyte antigen class I (HLA-I) and antigen-processing machinery (APM) [32]. The expression of HLA-I APM components in biopsies of melanoma responsive to anti-CTLA-4 therapy was higher and survival was longer than among nonresponders [33]. Activation of the immunoreceptor RIG-I led melanoma cells to sensitize to CD8+ T cells by inducing HLA-I APM expression. Another study has shown that higher expression of IL-1R was observed in nonresponsive melanoma patients to anti-PD-1 therapy [34].

Nerve growth factor receptor (NGFR^hi^) phenotype is involved not only in resistance to targeted therapy but also to immunotherapy [35,36]. NGFR expression was higher in nonresponders to anti-PD-1 or anti-PD-1 and anti-CTLA-4 combinations and was associated with low T cell infiltration in melanoma [36].

These findings indicated that melanoma cells induced different strategies to alter the IFN/STAT, PI3K/AKT, and WNT/β-catenin pathways, leading to survival under immunotherapy pressure. However, the interaction between these signaling pathways in melanoma resistance to immunotherapy is not yet understood and should be considered for further study.

Microbiota is a complex population of microorganisms. Recently, it was reported that gut microbiota is a main extrinsic modulator of the response to immunotherapy [37,38,39]. Combination treatment with fecal transfer and anti-PD-L1 enhanced the melanoma tumor control as compared to single treatment by increasing the CD8^+^ T cells in a mouse model [40]. This result indicates that gut microbiota contribute to the response to immunotherapy. Of note, it was reported that *Bifidobacterium* regulates the response to anti-PD-L1 treatment by activating DCs and then activating T cells. The gut microbiome of responders to immune checkpoint inhibitors was enriched for *Bacteroides thetaiotamicron*, *Dorea formicogenerans*, *Faecalibacterium prausnitzii*, and *Holdemania filiformis* [41]. Another study mentioned that higher diversity of the gut microbiota was observed in responding patients treated with anti-PD-1 therapy compared to nonresponders, but no significant changes in the oral microbiome were observed [42]. Moreover, high fecal diversity extended melanoma survival compared to the intermediate or low group. A more favorable *Ruminococcaceae* family enhanced the antitumor response by increasing antigen presentation and CD8^+^ T cells. In addition, metabolic and catabolic functions were enriched in the responsive and nonresponsive groups, respectively. *Bifidobacterium longum*, *Collinsella aerofaciens*, and *Enterococcus faecium* were enriched in melanoma in responders to anti-PD-1 treatment, while *Ruminococcus obeum* and *Roseburia intestinalis* were abundant in nonresponders [43]. Germ-free mice with fecal microbiome transplantation (FMT) from responders had decreased tumors and an increased response to anti-PD-L1 treatment compared to those transplanted with the fecal microbiome from nonresponders. A clinical trial (NCT03341143) was conducted to assess the effects of FMT in melanoma patients resistant to anti-PD-1 treatment whether alone or in combination with anti-CTLA-4 [44]. The combination of anti-PD-1 and FMT induced a response to immunotherapy in PD-1-resistant melanoma patients (6 of 15 patients) by increasing CD8^+^ T cells and decreasing IL-8-expressing myeloid cells.

## 3. miRNAs as Biomarkers to Predict the Response of Melanoma Patients to Immunotherapy

Immunotherapy is widely applied in the treatment of many cancers, but only a subset of patients derive benefit. Therefore, it is important to define who is suitable to receive immune checkpoint inhibitors. PD-L1 expression is the earliest and most promising predictive biomarker for anti-PD-1 therapy. Emerging evidence indicates that patients expressing high levels of PD-L1 experience an increased clinical benefit after treatment with anti-PD-1 agents. However, the definition of positive PD-L1 expression has been debated because different antibodies purified from different clones (clone 22C3 and clone 28-8) presented different evaluations. The cutoff value of PD-L1 staining was at least 5% and 1% of tumor cells by using clones 28-8 and clone 22C3, respectively [7,8]. Additionally, the expression of PD-L1 was dynamic during the course of clinical treatment [45,46]. The combination of PD-L1 status and the presence or absence of tumor-infiltrating lymphocytes has been considered as a promising biomarker for immunotherapy [47]. In addition, tumor mutation burden (TMB) has been considered to be a biomarker for immunotherapy [48,49,50]. Mutations in tumors may be translated to neoantigens that are recognized by T cells, leading to enhanced sensitivity to immunotherapy. Unfortunately, high cost and bioinformatic tools are the limitations of this assay. Moreover, the optimal cutoff value of TMB should be confirmed in different tumors. Several studies have been focused on microsatellite instability, mismatch-repair deficiency, somatic mutations, gut microbiome, human leukocyte antigen genotype, germline single-nucleotide polymorphisms, and circulating immune cells as predictive biomarkers for immunotherapy [45]. Recently, it was reported that greater aneuploidy in melanoma patients showed a poor response to immunotherapy [51]. These findings indicated that tumor aneuploidy could be considered as a potential prognostic marker for predicting the response of melanoma patients to immune checkpoint inhibitors.

Given that miRNAs have been widely studied as the biomarkers for many types of cancers, including melanoma, miR-199b-5p, miR-4488, and miR-524-5p could be considered as the predictive biomarkers for the response of melanoma patients to MAPK inhibitors [52,53]. In addition, miRNAs play a major role in the regulation of both the innate and adaptive immune systems. Therefore, it is possible to investigate miRNA-based biomarkers in response to immunotherapy. A previous study showed that adenosine deaminase acting on RNA 1 (ADAR1) was decreased in melanoma cells and that downregulation of ADAR1 supported melanoma cells to avoid tumor infiltrating lymphocyte-mediated killing by regulating intercellular adhesion molecule 1 (ICAM1) [54,55]. ICAM1 regulates the immune response through interaction with lymphocyte function-associated antigen-1 (LFA-1) that leads to T cell activation [56]. Overexpression of ADAR1 induced ICAM1 expression and blocking of ICAM1 reduced the functions of ADAR1 killing melanoma cells [55]. Overexpression of ADAR1 reduced the expression of miR-222 and miR-221, while ADAR1 knockdown increased miRNA levels. Moreover, miR-222, but not miR-221, directly targeted ICAM1. It indicated that ADAR1 regulated ICAM1 through miR-222. Importantly, miR-222 expression levels in tissues of nonresponders to ipilimumab (*n* = 23) were higher than those in the response group (*n* = 12). This result indicated that miR-222 could be considered as a prognostic marker for the response of melanoma to anti-CTLA-4 treatment (ipilimumab). Another study showed that the detection levels of five miRNAs, including let-7e, miR-99b, miR-125a, miR-125b, and miR-146b, in plasma could be predictive markers of the response of melanoma patients to ipilimumab and nivolumab [57]. Of note, higher expression of the miRNA cluster in relation to shorter progression-free survival and overall survival was found in melanoma patients treated with ipilimumab and nivolumab. Detection of miRNAs in exosomes promises to be a beneficial cancer biomarker method. Exosomes extracted from the serum of melanoma patients (54% of cutaneous, 22.7% of mucosal, and 23.3% of other subtype) treated with pembrolizumab or from nontreated serum were used to differentiate the expression of miRNAs between the two groups [58]. The exo-miRNA serum panel, including miR-532-5p and miR-106b, was decreased in melanoma patients treated with pembrolizumab (*n* = 57) compared with that from the nontreated group (*n* = 38). The area under the curve (AUC) values of miR-532-5p and miR-106b were 0.629 and 0.682, respectively, and the combination of both miRNAs reached an AUC value of 0.735. A high population of CD8+ T cells is a prognostic marker and is related to clinical results in many kinds of cancers [59]. It was reported that CD8+ T cells showed elevated miR-155 expression in a melanoma mouse model [60]. miR-155 expression in CD8+ T cells was increased after anti-PD-1 treatment in vivo and in situ. High expression of miR-155 or low expression of its target, PTPN2, was associated with higher survival of melanoma patients. This result indicated that the miR-155 level may be considered as a biomarker of the response to immunotherapy. A microarray was used to analyze 2560 different miRNAs in the serum from melanoma patients who responded to anti-PD-1 treatment (*n* = 3) and from nonresponders (*n* = 3) [61]. Thirteen miRNAs (miR-1972, miR-4502, miR-7110-5p, miR-3064-5p, miR-4459, miR-7107-5p, miR-1180-3p, miR-6799-5p, miR-7114-5p, miR-6849-5p, miR-4701-3p, miR-4462, and miR-6875-3p) and six miRNAs (miR-451a, miR-17-5p, miR-16-5p, miR-20a-5p, miR-106a-5p, and miR-1180-5p) were selected as the nonresponse and response markers, respectively. miR-1972 and miR-4502 and miR-16-5p, miR-17-5p, miR-20a-5p, and miR-451a were chosen for further evaluation because their highest expression and their targets were related to immunity. Serum expression of miR-16-5p, miR-17-5p, and miR-20a-5p was found to be higher in melanoma responding to anti-PD-1 treatment (*n* = 10) than in nonresponders (*n* = 23). In addition, the study reported that increased levels of miR-1972 and miR-4502 were detected in nonresponders. Another study investigated whether miR-615-3p, miR-1234-3p, and miR-4649-3p in serum were decreased in complete responders (*n* = 4) as compared to in partial responders (*n* = 4) among stage IV of melanoma patients; miR-3197 was used to distinguish stage III responders from nonresponders in pretreatment samples [62]. These findings highlighted the functions of miRNAs as prognostic biomarkers in response to immunotherapy in melanoma and are summarized in Table 1. In addition to melanoma, several miRNAs (miR-200b, miR-429, miR-93, miR-138-5p, miR-200, miR-27a, miR-424, miR-34a, miR-28, miR-106b, miR-193a-3p, miR-181a, miR-320d, miR-320c, and miR-320b) are also considered to be predictors of immunotherapy in lung cancer [63,64,65].

The above evidence demonstrates that miRNAs could be considered as effective predictors to indicate the response of cancers to immunotherapy. Circulating miRNAs would be a better choice due to noninvasive methods. However, a larger sample size should be conducted to investigate the sensitivity and specificity of these miRNAs.

## 4. Role of miRNAs in the Tumor Microenvironment

**Figure 2:**
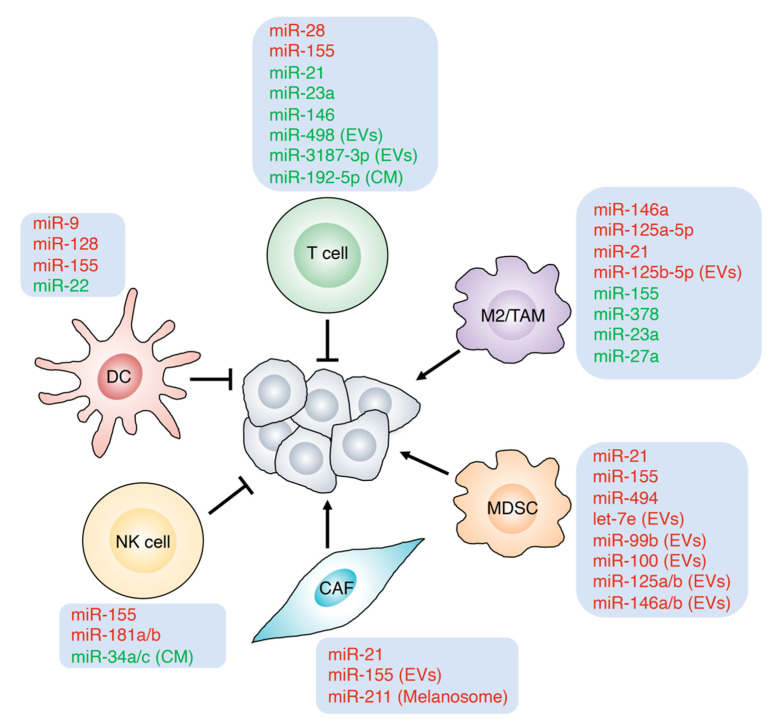
Regulator of miRNAs in microenvironment. miRNAs modulate the functions of immune cells in the tumor microenvironment. T cells, DCs, and NK cells suppress the progression of cancer, while MDSCs, M2/TAMs, and CAFs support the tumor development. M2/TAM: M2 macrophage/tumor-associated macrophage; MDSC: myeloid-derived suppressor cell; CAF: cancer-associated fibroblast; NK cell: natural killer cell; and DC: dendritic cell. Melanoma cells secrete miRNAs through extracellular vesicles (EVs) or conditioned medium (CM) to communicate and regulate the immune cells. miRNAs induce (red) or reduce (green) the functions of the immune cells included.

Tumor microenvironments are involved in the action of BRAF inhibitors including the efficacy and resistance [66,67]. It is indicated that the tumor microenvironment plays an important role in progression of melanoma. Cancer-associated fibroblasts (CAFs), DCs, T cells, macrophages, MDSCs, and NK cells are common cell populations in tumor immunity. Immune cells interact with cancer cells to regulate the tumor microenvironment including hypoxia and inflammation. In this section, we summarize the contribution of miRNAs to the major immune cells of the tumor microenvironment (Figure 2 and Table 2).

### 4.1. Inflammation

Inflammation is a key component of tumor development and induces pro-tumorigenic function by increasing angiogenesis, such as C-X-C motif ligand (CXCL) chemokines and matrix metalloproteinases (MMPs). It was reported that inflammation is an important indicator in diagnosis of the malignancy of cancers [68]. Inflammation mediated through JNK and NF-kB was reported to regulate miR-155 expression by binding to its promoter, indicating that miR-155 is an important miRNA involved in the interaction between cancer and inflammation [69]. MITF-M, an isoform of microphthalmia-associated transcription factor (MITF), is an important transcription factor that regulates the expression of a variety of genes, such as tyrosinase (TYR), Melan-A/Mart-1 (MLANA), or pMel17/gp100 (SILV), that are recognized by cytolytic T lymphocytes (CTL) [70]. Stimulation of inflammation reduced the expression of MITF-M through upregulation of miR-155, leading to the immune escape of melanoma [70]. miR-146 is a negative modulator of NF-κB signaling by targeting TNFR-associated factor 6 (TRAF6) and Il-1R-associated kinase 1/2 (IRAK1/2) [71,72]. Increased miR-155 expression produced inflammation in miR-146a-deficient mice through upregulation of NF-κB activity [73]. miR-9 was increased when exposed to pro-inflammatory signals in human polymorphonuclear neutrophils (PMN) and monocytes through activation of the MyD88-and NF-κB-dependent signaling pathway [74]. It indicated that miR-155, miR-146, and miR-9 played a central role in inflammatory response.

### 4.2. Cancer-Associated Fibroblasts (CAFs)

Normal fibroblasts play a key role in the inhibition of the early stage of melanoma, but this function is lost by changes in the tumor microenvironment. CAFs are activated fibroblasts that are abundant cell types in the tumor microenvironment. CAFs modulate cancer behavior including growth, metastasis, and therapeutic resistance. Inactivation of miR-21 or overexpression of its target, SMAD family member 7 (SMAD7), blocked the TGF-β1-induced CAF formation [75]. Melanosomes containing miR-211 educate primary fibroblasts to become CAFs [76]. Melanosomal miR-211 enhanced the formation of CAFs by targeting the tumor suppressor insulin-like growth factor 2 receptor (IGF2R) and then activated the MAPK/ERK pathway. miR-155 and miR-210 were detected in human melanoma-derived exosomes by using immuno-biochip [77]. Exosomal miR-155 and miR-210 were delivered into human adult dermal fibroblasts, leading to modified metabolic activities of the recipient cells. Moreover, another study reported that melanoma cells secreted miR-155-carrying exosomes to differentiate fibroblasts into proangiogenic CAFs, thereby inducing proliferation and migration in vitro and tumor progression in vivo [78]. Suppressor of cytokine signaling 1 (SOCS1) is a direct target of miR-155 in fibroblast cells. Moreover, exosomal miR-155 activates the JAK2/STAT3 signaling pathway and increases the expression levels of matrix metallopeptidase 9 (MMP9) and proangiogenic cytokines, including vascular endothelial growth factor A (VEGFA) and fibroblast growth factor 2 (FGF2).

### 4.3. Dendritic Cells (DCs)

DCs are the key regulators of the antitumor immune response and induce the activation and differentiation of naïve T cells by presenting antigens to naïve T cells. miR155-deficient DCs failed to activate T cells by reducing antigen presentation and cytokine production [79,80]. The p38 MAPK pathway plays a key role in regulating the maturation of DC cells via IL-10 [81]. Inhibition of miR-22 or overexpression of miR-128 enhances the tumor-suppressing role of DC cells by targeting p38 [82,83]. Melanoma tumor growth was decreased in DC-inhibited miR-22 or DC-overexpressed miR-128. Elevated expression of miR-9 was found both in bone marrow-derived dendritic cells (BMDCs) and conventional DC1s (cDC1s), modulators of the antitumor immune response through NF-κB signaling [84]. Overexpression of miR-9 in BMDCs not only promotes DC activation and functions by targeting polycomb group ring finger 6 (PCGF6), an inhibitor of DC activation, but also activates CD4^+^ and CD8^+^ T cells. Additionally, miR-9 overexpression in DCs reduced tumor progression in a melanoma mouse model. miR-192-5p- and miR-148a-3p-derived hypoxic melanoma cells were transferred into DCs by the Cx43 channel and miR-192-5p was delivered to both DCs and T cells to inhibit their functions [85].

### 4.4. T Cells

Several studies have focused on the roles of miRNAs in melanoma response to immunotherapy by impacting on tumor cells or immune cells. CD8+ T cells express antitumor functions and, therefore, are a top candidate for immunotherapy. It has been reported that CTLA-4, PD-1, and PDL-1 are regulated by miRNAs [86]. Growing evidence has revealed the functions of miRNAs in T cells through the regulation of key pathways and molecules related to the activation of T cells. miR-23a acts as a negative mediator of CD8+ T cells [87]. miR-23a directly targets B lymphocyte-induced maturation protein-1 (BLIMP-1), a key transcription regulator of T cell function, to attenuate the antitumor response of cytotoxic T cells by reducing granzyme B, and IFN-γ. It has been reported that PD-1 is a target of miR-28 in T cells [88]. Low levels of miR-28 increased the expression of inhibitory receptors, including PD-1, T cell immunoglobulin domain and mucin domain 3 (TIM3), and B and T lymphocyte attenuator (BTLA), which enable tumor cells to evade immune control in a melanoma mouse model. Importantly, high expression of miR-28 rescued the exhausted T cells by recovering the ability of T cells to induce cytokine production, including IL-2 and TNF-α. miR-21-deficient mice had reduced tumor size as compared to that of wild-type mice [89]. Moreover, tumor-associated macrophages (TAMs) shifted to the M1 phenotype in miR-21-/- mice and repaired the function of T cells to induce proinflammatory cytokines and cytolytic granules. miR-21 negatively regulated the IFN-γ pathway by directly targeting STAT1 and indirectly targeting JAK2. The combination of miR-21-/- TAMs and anti-PD1 significantly reduced tumor growth compared to the single treatment. miR-146 expression is known to be increased in melanoma tissues as compared to that in nevi and healthy tissues [90]. miR-146a-/- mice had longer survival than wild-type mice by inducing IFN-γ-producing T cells through activation of STAT1. Moreover, elevated PD-L1 levels were observed in miR-146a-/- mice and in melanoma cells treated with IFN-γ. The combination of anti-miR-146a and anti-PD-1 reduced melanoma tumors and prolonged the survival of the mice compared to treatment with anti-PD-1 alone. miR-146a-/- mice treated with anti-PD-1 induced immune-related adverse events (irAEs) as opposed to the wild-type group [91]. It has been shown that a better clinical response to immune checkpoint inhibitors correlates with the induction of irAEs [92,93]. Enhanced accumulation of CD4+ and CD8+ T cells and increased inflammatory cytokines are involved in irAEs [94]. More activated T cells, CD4+ T cells, and neutrophil recruitment have been observed in miR-146a-/- mice treated with anti-PD-1. It has been demonstrated that miR-155 is a core modulator of the immune response [79]. miR-155 expression is high in T cells, B cells, DCs, and macrophages [95]. Overexpression of miR-155 in CD8+ T cells enhances the antitumor response by reducing the expression of signal transducer and activator of transcription 5 (STAT5), Src homology-2 domain-containing inositol 5-phosphatase 1 (SHIP1), SOCS1, and PTPN2 [96]. Loss of miR-155 in T cells leads to reduced antitumor immunity in a melanoma mouse model by decreasing the activated T cell response and increasing the population of myeloid cells [97,98,99]. Moreover, miR-155 T cell-conditional knockout mice exhibit enhanced tumor growth and reduced IFN-γ-expressing CD4+ and CD8+ T cells [98]. Additionally, high expression of miR-155 prolongs the survival of skin cutaneous melanoma [97]. Moreover, miR-155 acts as a tumor suppressor in melanoma [100,101,102]. Interestingly, immunotherapy rescues the deficient antitumor response caused by the lack of miR-155 in T cells by reducing miR-155 target genes, including SOCS1, BTB domain and CNC homolog 1 (BACH1), CCAAT enhancer binding protein beta (CEBPB), and interleukin 7 receptor (IL7R) [98]. It has been reported that T cell receptor signaling induces NF-κB and activator protein 1 (AP-1), which binds to the miR-155 promoter to increase its expression [103,104]. A previous study demonstrated that miR-498 and miR-3187-3p in melanoma-derived exosomes reduces the functions of CD8+ T cells by targeting IFN-α and protein tyrosine phosphatase receptor type C (PTPRC), the coding gene for CD45, respectively [105]. Hypoxic melanoma cells deliver miR-192-5p to cytotoxic T cells via Connexin-43 (Cx43)-constituted gap junctions to reduce the T cell functioning [85].

### 4.5. Macrophages

Several studies have reported that miRNAs modulate macrophage polarization. M1 macrophages present antitumor activities, while M2 macrophages induce tumor growth. In addition, another subtype of macrophages is TAMs, which promote tumorigenesis. A high population of TAMs has been found to be related to poor prognosis and survival in melanoma patients. miR-155 induces the activation of M1 macrophages by targeting SOCS1, B-cell lymphoma-6 protein (BCL6), and interleukin 13 receptor alpha 1 (IL13Rα1), and miR-378 induces the activation of M1 macrophages by targeting AKT serine/threonine kinase 1 (AKT1) [106,107,108,109]. Conversely, miR-146a attenuates the differentiation of M1 macrophages by suppressing the expression of interleukin 1 receptor-associated kinase 1 (IRAK1) and TNF receptor-associated factor 6 (TRAF6) [71]. The miR-23a/27a/24-2 cluster is an important mediator of macrophage polarization [110]. miR-23a and miR-27a reduced M2 differentiation by targeting JAK1/STAT6 and IRF4/PPAR-γ, respectively. miR-125a-5p promotes the differentiation of macrophages to the M2 phenotype by downregulating Kruppel-like factor 13 (KLF13) [111]. It has been reported that miR-125b-5p packaged in melanoma exosomes induces a TAM phenotype by downregulating lysosomal acid lipase A (LIPA) [112]. miR-21 is an oncogene in melanoma and other cancers. miR-21 increased melanoma development by reducing IFN action [113]. Inhibition of miR-21 in TAMs reduced tumor growth by inducing proinflammatory immune responses, including TNF, CXCL10, and CXCL9 [114]; this activates CD8^+^ T cells and causes the secretion of cytokines and chemokines, including IL-12 and CXCL10. In a mouse model of melanoma, miR-21, miR-29a, miR-142-3p, miR-181a, and miR-223 have been found to be increased in tumor-infiltrating myeloid cells (mature myeloid cells and macrophages) [115]. Overexpression of miR-21 and miR-29a in macrophages cocultured with melanoma cells promoted the melanoma cell proliferation and angiogenesis.

**Table 2:** miRNAs involved in tumor immunity.

miRNA	Functions in Tumor Immunity	Target Genes	Response to Immunotherapy	Ref.
miR-21	Reduce activity of T cells, and promote TAM polarization, MDSCs and CAFs	STAT1, SMAD7	Reduce the response to anti-PD-1	[75,89,114]
miR-155	Enhance antitumor response of CD8^+^ T cells, activation of M1 macrophages and maturation of DCs Promote the functions of MDSCs Secrete via melanoma EVs to induce differentiation of CAFs Positive regulator of NK cells	STAT5, SHIP1, SOCS1, PTPN2, BACH1, CEBPB, IL7R, BCL6, IL13Rα1, PTEN, KPC1, DC115		[16,78,79,80,95,96,97,99,106,107,108,116,117]
miR-211	Secrete from melanosome to educate primary fibroblasts to become CAFs	IGF2R		[76]
miR-9	Promote activation and functions of DCs	PCGF6		[84]
miR-128	Enhance anti-tumor response of DCs	p38		[83]
miR-22	Reduce the tumor-suppressing role of DCs	p38		[82]
miR-28	Rescue exhaustive T cells	PD-1		[88]
miR-23a	Negative modulator of CD8^+^ T cells Inhibit M2 polarization	BLIMP-1, JAK1, STAT6		[87,110]
miR-146	Reduce immune activation		Reduce the response to anti-PD-1	[90,91]
miR-498 miR-3187-3p	Secrete via melanoma EVs to reduce T cell responses	IFN-γ, PTPRC		[105]
miR-192-5p	Regulator of melanoma cells to reduce the cytotoxicity of T cells			[85]
miR-125a-5p	Promote M2 phenotype	KLF13		[111]
miR-125b-5p	Secrete from melanoma EVs to induce TAM phenotypes	LIPA		[112]
miR-378	Induce activation of M1 macrophages	AKT1		[109]
miR-27a	Inhibit M2 polarization	IRF4, PPAR-γ		[110]
miR-494	Enhance the functions of MDSCs	PTEN		[118]
let-7e miR-99b miR-100 miR-125a/b miR-146a/b	Secrete from melanoma EVs to induce the polarization and functions of MDSCs			[57]
miR-181a/b	Induce development and functions of NK cells			[119]
miR-34a/c	Induce from melanoma cells to negatively regulate the cytolytic activity of NK cells	ULBP2		[120]

### 4.6. Myeloid-Derived Suppressor Cells (MDSCs)

MDSCs are a heterogenous cell population that includes myeloid progenitor cells and immature myeloid cells. MDSCs develop during tumor progression, present immunosuppressive functions, and reduce the activation of T cells. miR-155 and miR-21 induce the expansion of MDSCs by targeting SHIP-1 and PTEN, respectively [116]. Another study demonstrated that miR-155 is required for the accumulation and differentiation of MDSCs by downregulating SOCS1 [117]. miR-494 expression was upregulated in MDSCs from a melanoma-bearing mouse model compared to the control by TGF-β1 [118]. It was found that miR-494 regulates the activity of MDSCs by targeting PTEN, which, in turn, activates the AKT signaling. Extracellular vesicles (EVs) from melanoma convert myeloid cells to MDSCs to inhibit T cell functions by increasing IL-6 and CCL2 [57]. In melanoma patients, exosome carrying miR-146a, miR-155, miR-125b, miR-100, let-7e, miR-125a, miR-146b, and miR-99b are involved in the polarization and functions of MDSCs [57]. The levels of these miRNAs were increased in plasma samples of melanoma patients as compared to healthy donors. The study indicated that these miRNAs might be considered markers for the status of MDSCs in the tumor site.

### 4.7. Natural Killer Cells (NK Cells)

In the tumor immunity, NK cells suppress the growth of tumor cells by producing immunostimulatory cytokines. Several miRNAs were investigated to be involved in the functions of NK cells. miR-181a/b induces the development and functions of NK cells by producing IFN-γ. Knockdown of miR-181a/b increases the expression of Nemo-like kinase (NLK), a negative regulator of the Notch pathway in NK cells [119]. Another study reported that miR-155 is a positive regulator of IFN-γ in NK cells by suppressing SHIP1, a negative regulator of IFN-γ [16]. miR-34a and miR-34c target UL16 binding protein 2 (ULBP2) to decrease the cytolytic activity of NK cells [120]. Overexpression of miR-34a and miR-34c in melanoma cells downregulates the ability of NK cells to recognize melanoma cells by targeting ULBP2. The interaction between NKG2D, a receptor of NK cells and cytotoxic T lymphocytes (CTLs), and ULBP2 (NKG2D ligand) expressed in tumor cells, allows NK cells to kill the tumor cells. Antigen processing 1 (TAP1) and HLA-A are the components required for the function of T cells. High expression of TAP1 and HLA-A increased the survival probability of melanoma patients [121]. miR-200a-5p overexpressed in melanoma cells increases their sensitivity to being killed by NK cells by directly targeting TAP1, followed by the reduced expression of HLA-I. Importantly, melanoma patient survival in the high miR-200a-5p expression group was shorter than that in the low miR-200a-5p group.

## 5. Conclusions and Future Aspects

Multiple strategies have been developed for the treatment of melanoma. Although immunotherapy has the ability to prolong the survival of melanoma patients, resistance is a limitation. Elucidation of the resistance mechanism to immunotherapy is required to resolve the issue. In addition, a good predictor of the response to immune checkpoint inhibitors would play an important role in deciding the suitable treatment for melanoma patients. Recently, many studies have started to focus on the function of miRNAs as diagnostic and prognostic markers in melanoma, as well as in other cancers. However, the understanding about utilizing miRNAs as a prognostic marker for immunotherapy in melanoma is limited due to sample size and number of the studies. In addition, classification of patients into complete response, partial response, and progressive disease should be included in future study to explore the potential role of miRNAs. Of note, miRNAs are stable and easily detected in plasma or serum that indicates detection expression levels of miRNAs are a non-invasive approach to apply in the biomarker field. In this regard, combination between expression levels of miRNAs and PD-L1 may be considered as an effective predictor of immunotherapy. As recapitulated in this review, miRNAs participate in regulating tumor development and the immune response. Overexpression or silencing the expression of miRNAs could impact on therapeutic treatment of melanoma including response and resistance. As mentioned above, one miRNA is able to target multiple genes related to immune checkpoint mechanisms and functions of the key immune cells in the tumor microenvironment, indicating that miRNAs could imitate the combination treatment and regulate diverse mechanisms. Therefore, it is reasonable to consider miRNAs as a therapeutic partner of immunotherapy. However, ongoing study should be focused on melanoma patients to figure out the suitable strategy for applications of miRNAs and fill in the knowledge gap. Importantly, miR-155 and miR-21 are the important miRNAs that regulate the tumor environment in melanoma. Of note, miR-155 plays dual roles in tumor immunity, including immunostimulation and immunosuppression, through different mechanisms. In addition, miR-155 acts as a tumor suppressor in melanoma, ovarian cancer, and gastric cancer or an oncogene in breast cancer, colon cancer, pancreatic cancer, oral squamous cell carcinoma, and nasopharyngeal carcinoma [122]. These findings demonstrate that the functions of miR-155 are distinct in different tumor types. Therefore, it is necessary to deeply understand the functions of miR-155 in tumor immunity before considering its clinical applications. In summary, miRNAs could be considered important partners of immunotherapy or therapeutic targets for the treatment of cancers.

## Figures and Tables

**Table 1:** miRNAs as predicted biomarkers for immunotherapy.

miRNA	Sample Source	Expression	Target Genes	Ref.
miR-222	Tissue	Low in clinical benefit melanoma tissues received anti-CTLA-4 (ipilimumab)	ICAM1	[55]
let-7e miR-99b miR-125a miR-125b miR-146b	Plasma EVs	High expression of miRNA cluster reduced the overall survival and progression-free survival of the patients treated with anti-CTLA-4 (ipilimumab) and anti-PD-1 (nivolumab)		[57]
miR-106b miR-532-5p	Serum	Decrease in melanoma treated with anti-PD-1 (pembrolizumab)		[58]
miR-155	Peripheral blood	Increase after treatment with anti-PD-1	PTPN2	[60]
miR-16-5p miR-17-5p miR-20a-5p	Serum	High in serum from melanoma responded to anti-PD-1 (nivolumab or pembrolizumab)		[61]
miR-1972 miR-4502	Serum	Increase in non-responders treated with anti-PD-1 (nivolumab or pembrolizumab)		[61]
miR-615-3p miR-1234-3p miR-4649-3p	Serum	Decrease in responders received anti-PD-1 (nivolumab or pembrolizumab) and anti-CTLA-4 (ipilimumab) or combination of ipilimumab and nivolumab		[62]

## Abbreviations

FDA	Food and Drug Administration,
MDSCs	myeloid-derived suppressor cells,
JAK1	Janus kinase 1,
JAK2	Janus kinase 2,
B2M	beta-2-microglobulin,
MHC-I	major histocompatibility complex class 1,
STAT1	signal transducer and activator of transcription 1,
IFNGR1	interferon gamma receptor 1,
PTPN2	protein tyrosine phosphatase non-receptor type 2,
APLNR	apelin receptor,
PBRM1	polybromo 1,
ARID2	AT-rich interaction domain 2,
BRD7	bromodomain containing 7,
USP22	ubiquitin-specific peptidase 22,
PTEN	phosphatase and tensin homolog,
CDKN2A	cyclin-dependent kinase inhibitor 2A,
SK1	sphingosine kinase-1,
HLA-I	human leukocyte antigen class I,
APM	antigen-processing machinery,
NGFR	nerve growth factor receptor,
FMT	fecal microbiome transplantation,
TMB	tumor mutation burden,
ADAR1	adenosine deaminase acting on RNA 1,
ICAM1	intercellular adhesion molecule 1,
AUC	area under curve,
CAFs	cancer-associated fibroblasts,
BLIMP-1	B lymphocyte-induced maturation protein-1,
TIM3	T cell immunoglobulin domain and mucin domain 3,
BTLA	B and T lymphocyte associated,
TAMs	tumor-associated macrophages,
irAEs	immune-related adverse events,
DCs	dendritic cells,
STAT5	signal transducer and activator of transcription 5,
SHIP1	Src homology-2 domain-containing inositol 5-phosphatase 1,
SOCS1	suppressor of cytokine signaling 1,
BACH1	BTB domain and CNC homolog 1,
CEBPB	CCAAT enhancer binding protein beta,
IL7R	interleukin 7 receptor,
NF-κB	nuclear factor kappa B,
AP-1	activator protein 1,
PTPRC	protein tyrosine phosphatase receptor type C,
BCL6	B-cell lymphoma-6 protein,
IL13Rα1	interleukin 13 receptor alpha1,
AKT1	AKT serine/threonine kinase 1,
IRAK1	interleukin 1 receptor-associated kinase 1,
TRAF6	TNF receptor-associated factor 6,
STAT6	signal transducer and activator of transcription 6,
IRF4	interferon regulatory factor 4,
PPAR-γ	peroxisome proliferator activated receptor gamma,
KLF13	Kruppel-like factor 13,
LIPA	lysosomal acid lipase A,
EVs	extracellular vesicles,
SMAD7	SMAD family member 7,
IGF2R	insulin-like growth factor 2 receptor,
VEGFA	vascular endothelial growth factor A,
FGF2	fibroblast growth factor 2,
NLK	Nemo-like kinase,
ULBP2	UL16 binding protein 2,
TAP1	antigen processing 1,
HLA-A	major histocompatibility complex class I, A,
BMDCs	bone marrow-derived dendritic cells,
cDC1s	conventional DC1s,
CXCL	C-X-C motif ligand,
MMPs	matrix metalloproteinases,
JNK	c-Jun N-terminal kinase,
MITF	microphthalmia-associated transcription factor,
TYR	tyrosinase,
MLANA	Melan-A/Mart-1,
CTL	cytolytic T lymphocytes,
TRAF6	TNFR-associated factor 6,
IRAK1/2	Il-1R-associated kinase 1/2.
